# Supplementary material for: Urinary prostaglandin metabolites as biomarkers for human labour: Insights into future predictors
Source: PLoS One. 2025 Jul 14;20(7):e0315484. doi: 10.1371/journal.pone.0315484 (PMC12258607; doi:10.1371/journal.pone.0315484)
Supplement: S2 Appendix — (PDF) [file pone.0315484.s002.pdf]

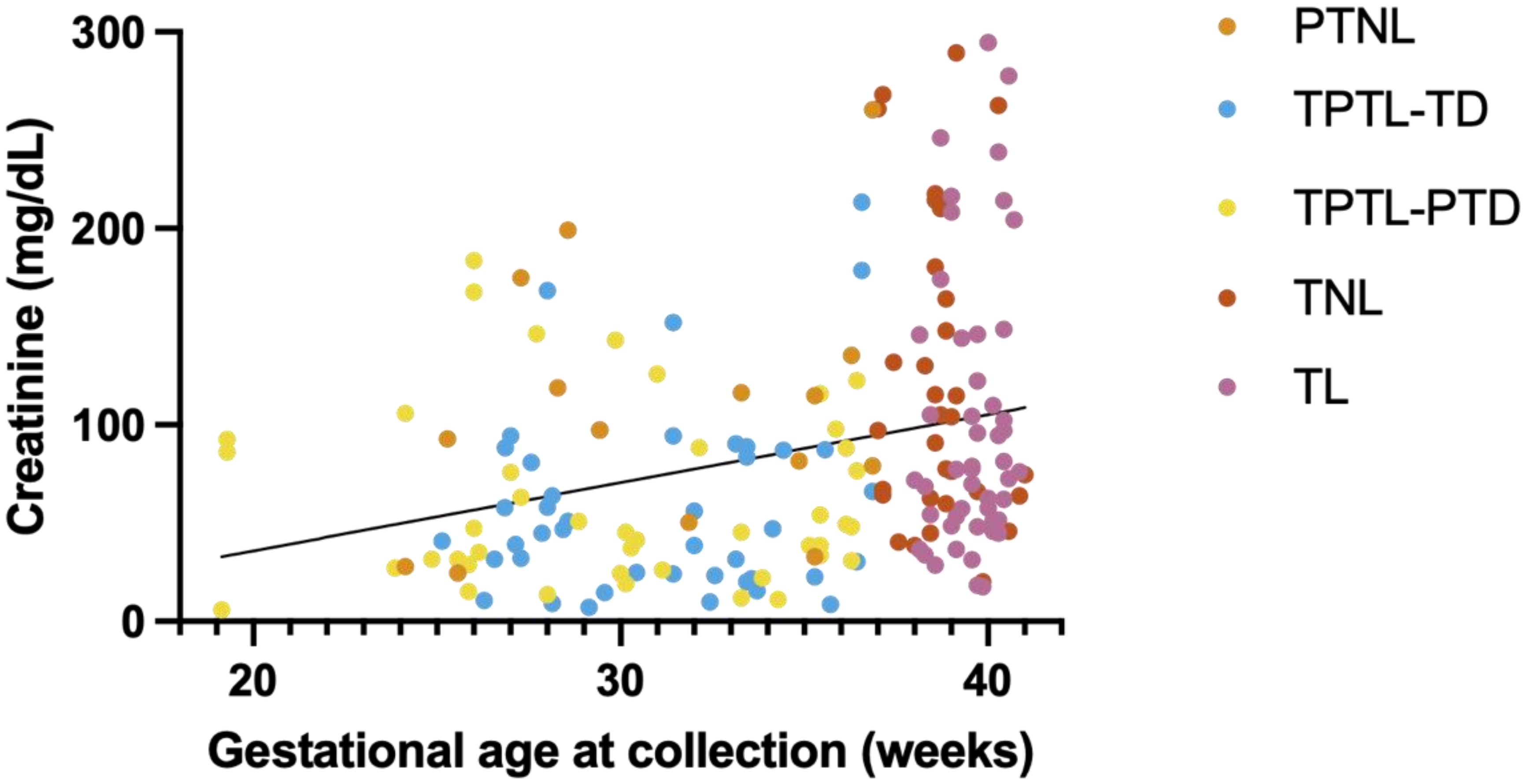

Supplemental Fig 1. Urinary creatinine levels throughout pregnancy by gestational age at sample collection. Analyzed by simple linear regression: $R^2 = 0.080$, $\beta = 3.47$, $p<0.001$. Regression equation represents all samples grouped together.
