# Supplementary material for: Urinary prostaglandin metabolites as biomarkers for human labour: Insights into future predictors
Source: PLoS One. 2025 Jul 14;20(7):e0315484. doi: 10.1371/journal.pone.0315484 (PMC12258607; doi:10.1371/journal.pone.0315484)
Supplement: S3 Appendix — (DOCX) [file pone.0315484.s003.docx]

UCSD Lipidomics Core eicosanoid panel

| **Eicosanoid** | **Full Name** | **Systematic Name** | **LIPID MAPS ID** |
| --- | --- | --- | --- |
| 6k PGF1a | 6-keto-PGF1α | 6-oxo-9S,11R,15S-trihydroxy-13E-prostenoic acid | LMFA03010001 |
| TxB2 | TXB2 | 9S,11,15S-trihydroxy-thromboxa-5Z,13E-dien-1-oic acid | LMFA03030002 |
| PGF2a | PGF2α | 9S,11R,15S-trihydroxy-5Z,13E-prostadienoic acid | LMFA03010002 |
| PGE2 | PGE2 | 9-oxo-11R,15S-dihydroxy-5Z,13E-prostadienoic acid | LMFA03010003 |
| PGD2 | PGD2 | 9S,15S-dihydroxy-11-oxo-5Z,13E-prostadienoic acid | LMFA03010004 |
| 11b PGF2a | 11β-PGF2α | 9S,11S,15S-trihydroxy-5Z,13E-prostadienoic acid | LMFA03010036 |
| TXB1 | TXB1 | 9S,11,15S-trihydroxy-thrombox-13E-enoic acid | LMFA03030008 |
| PGF1a | PGF1a | 9S,11R,15S-trihydroxy-13E-prostaenoic acid | LMFA03010137 |
| PGE1 | PGE1 | 9-oxo-11R,15S-dihydroxy-13E-prostaenoic acid | LMFA03010134 |
| PGD1 | PGD1 | 9-oxo-15S,19-dihydroxy-5Z,8(12),13E-prostatrienoic acid | LMFA03010132 |
| TXB3 | TXB3 | 9S,11,15S-trihydroxy-thromboxa-5Z,13E,17Z-trien-1-oic acid | LMFA03030006 |
| PGF3a | PGF3a | 9S,11R,15S-trihydroxy-5Z,13E,17Z-prostatrienoic | LMFA03010138 |
| PGE3 | PGE3 | 9-oxo-11R,15S-dihydroxy-5Z,13E,17Z-prostatrienoic acid | LMFA03010135 |
| PGD3 | PGD3 | 9S,15S-dihydroxy-11-oxo-5Z,13E,17Z-prostatrienoic acid | LMFA03010142 |
| dihomo PGF2a | 1a,1b-dihomo-PGF2a | 1a,1b-dihomo-9S,11R,15S-trihydroxy-5Z,13E-prostadienoic acid | LMFA03010157 |
| 15k PGF2a | 15-keto-PGF2α | 9S,11R-dihydroxy-15-oxo-5Z,13E-prostadienoic acid | LMFA03010026 |
| 15k PGE2 | 15-keto-PGE2 | 9,15-dioxo-11R-hydroxy-5Z,13E-prostadienoic acid | LMFA03010030 |
| dhk PGF2a | 13,14-dihydro-15-keto-PGF2α | 9S,11S-dihydroxy-15-oxo-5Z-prostenoic acid | LMFA03010027 |
| dhk PGE2 | 13,14-dihydro-15-keto-PGE2 | 9,15-dioxo-11R-hydroxy-5Z-prostenoic acid | LMFA03010031 |
| dhk PGD2 | 13,14-dihydro-15-keto-PGD2 | 11,15-dioxo-9S-hydroxy-5Z-prostenoic acid | LMFA03010022 |
| bicyclo PGE2 | bicyclo-PGE2 | 9,15-dioxo-5Z-prostaenoic acid-cyclo[11S,16] | LMFA03010034 |
| 20oh PGF2a | 20-hydroxy-PGF2a | 9S,11S,15S,20-tetrahydroxy-5Z,13E-prostadienoic acid | LMFA03010029 |
| 20oh PGE2 | 20-hydroxy-PGE2 | 9-oxo-11R,15S,20-trihydroxy-5Z,13E-prostadienoic acid | LMFA03010014 |
| 2,3 dinor 11b PGF2a | 2,3-dinor 11β-PGF2a | 9S,11S,15S-trihydroxy-2,3-dinor-5Z,13E-prostadienoic acid | LMFA03010011 |
| Tetranor-PGFM | Tetranor-PGFM | 9S,11R-dihydroxy-15-oxo-2,3,4,5-tetranor-prostan-1,20-dioic acid | LMFA03010139 |
| Tetranor-PGEM | Tetranor-PGEM | 11R-hydroxy-9,15-dioxo-2,3,4,5-tetranor-prostan-1,20-dioic acid | LMFA03010032 |
| tetranor 12-HETE | Tetranor-12(R)-HETE | 8R-hydroxy-4Z,6E,10Z-hexadecatrienoic acid | LMFA01050143 |
| PGK2 | PGK2 | 9,11-dioxo-15S-hydroxy-5Z,13E-prostadienoic acid | LMFA03010023 |
| 12-HHTrE | 12S-HHTrE | 12S-hydroxy-5Z,8E,10E-heptadecatrienoic acid | LMFA03050002 |
| 11-HETE | 11S-HETE | 11S-hydroxy-5Z,8Z,11E,14Z-eicosatetraenoic acid | LMFA03060003 |
| 11-HEPE | (+/-) 11-HEPE | (+/-)-11-hydroxy-5Z,8Z,12E,14Z,17Z-eicosapentaenoic acid | LMFA03070030 |
| 13 HDoHE | (+/-)-13-HDoHE | (+/-)-13-hydroxy-4Z,7Z,10Z,14E,16Z,19Z-docosahexaenoic acid | LMFA04000029 |
| PGA2 | PGA2 | 9-oxo-15S-hydroxy-5Z,10Z,13E-prostatrienoic acid | LMFA03010035 |
| PGB2 | PGB2 | 15S-hydroxy-9-oxo-5Z,8(12),13E-prostatrienoic acid | LMFA03010018 |
| PGJ2 | PGJ2 | 11-oxo-15S-hydroxy-5Z,9,13E-prostatrienoic acid | LMFA03010019 |
| 15d PGD2 | 15-deoxy-delta-12,14-PGD2 | 9S-hydroxy-11-oxo-5Z,12E,14E-prostatrienoic acid | LMFA03010051 |
| 15d PGJ2 | 15-deoxy-δ-12,14-PGJ2 | 11-oxo-5Z,9,12E,14E-prostatetraenoic acid | LMFA03010021 |
| 5-iso PGF2a VI | (+/-) 5-iPF2alpha-VI | 5,9S,11R-trihydroxy-6E,14Z-prostadienoic acid-cyclo[8S,12R] | LMFA03110011 |
| 8-iso PGF2a III | 8-iso-PGF2a | 9S,11R,15S-trihydroxy-5Z,13E-prostadienoic acid-cyclo[8S,12R] | LMFA03110001 |
| 9-HETE | 9-HETE | 9-hydroxy-5Z,7E,11Z,14Z-eicosatetraenoic acid | LMFA03060089 |
| 9-HEPE | (+/-) 9-HEPE | (+/-)-9-hydroxy-5Z,7E,11Z,14Z,17Z-eicosapentaenoic acid | LMFA03070029 |
| 8 HDoHE | (+/-)-8-HDoHE | (+/-)-8-hydroxy-4Z,6E,10Z,13Z,16Z,19Z-docosahexaenoic acid | LMFA04000026 |
| 16 HDoHE | (+/-)-16-HDoHE | (+/-)-16-hydroxy-4Z,7Z,10Z,13Z,17E,19Z-docosahexaenoic acid | LMFA04000031 |
| 20 HDoHE | (+/-)-20-HDoHE | (+/-)-20-hydroxy-4Z,7Z,10Z,13Z,16Z,18E-docosahexaenoic acid | LMFA04000033 |
| LTB4 | LTB4 | 5S,12R-dihydroxy-6Z,8E,10E,14Z-eicosatetraenoic acid | LMFA03020001 |
| 20oh LTB4 | 20-hydroxy LTB4 | 5S,12R,20-trihydroxy-6Z,8E,10E,14Z-eicosatetraenoic acid | LMFA03020018 |
| 20cooh LTB4 | 20-carboxy-LTB4 | 5S,12R-dihydroxy-6Z,8E,10E,14Z-eicosatetraene-1,20-dioic acid | LMFA03020016 |
| 5,6-diHETE | 5S,6R-DiHETE | 5S,6R-dihydroxy-7E,9E,11Z,14Z-eicosatetraenoic acid | LMFA03060017 |
|  | 5S,6S-DiHETE | 5S,6S-dihydroxy-7E,9E,11Z,14Z-eicosatetraenoic acid | LMFA03060018 |
| 5,12-diHETE | 5,12-DiHETE | 5,12-dihydroxy-6,8,10,14-eicosatetraenoic acid | LMFA03060052 |
| 12oxo LTB4 | 12-oxo-LTB4 | 5S-hydroxy-12-keto-6Z,8E,10E,14Z-eicosatetraenoic acid | LMFA03020024 |
| LTC4 | LTC4 | 5S-hydroxy,6R-(S-glutathionyl),7E,9E,11Z,14Z-eicosatetraenoic acid | LMFA03020003 |
| LTD4 | LTD4 | 5S-hydroxy-6R-(S-cysteinylglycinyl)-7E,9E,11E,14Z-eicosatetraenoic acid | LMFA03020006 |
| LTE4 | LTE4 | 5S-hydroxy,6R-(S-cysteinyl),7E,9E,11Z,14Z-eicosatetraenoic acid | LMFA03020002 |
| 11t LTC4 | 11-trans-LTC4 | 5S-hydroxy-6R-(S-glutathionyl)-7E,9E,11E,14Z-eicosatetraenoic acid | LMFA03020020 |
| 11t LTD4 | 11-trans-LTD4 | 5S-hydroxy-6R-(S-cysteinylglycinyl)-7E,9E,11E14Z-eicosatetraenoic acid | LMFA03020021 |
| 11t LTE4 | 11-trans-LTE4 | 5S-hydroxy-6R-(S-cysteinyl)-7E,9E,11E14Z-eicosatetraenoic acid | LMFA03020022 |
| 5-HETE | 5S-HETE | 5S-hydroxy-6E,8Z,11Z,14Z-eicosatetraenoic acid | LMFA03060002 |
| 5-HEPE | (+/-) 5-HEPE | (+/-)-5-hydroxy-6E,8Z,11Z,14Z,17Z-eicosapentaenoic acid | LMFA03070027 |
| 7 HDoHE | (+/-)-7-HDoHE | (+/-)-7-hydroxy-4Z,8E,10Z,13Z,16Z,19Z-docosahexaenoic acid | LMFA04000025 |
| 4 HDoHE | (+/-)-4-HDoHE | (+/-)-4-hydroxy-5E,7Z,10Z,13Z,16Z,19Z-docosahexaenoic acid | LMFA04000024 |
| 9-HOTrE | 9(S)-HOTrE | 9S-hydroxy-10E,12Z,15Z-octadecatrienoic acid | LMFA02000024 |
| 5-HETrE | 5(S)-HETrE | 5S-hydroxy-6E,8Z,11Z-eicosatrienoic acid | LMFA03050005 |
| 5,15-diHETE | 5S,15S-DiHETE | 5S,15S-dihydroxy-6E,8Z,11Z,13E-eicosatetraenoic acid | LMFA03060010 |
| 6R,15R-LXA4 | 5S,6R-LipoxinA4 | 5S,6R,15S-trihydroxy-7E,9E,11Z,13E-eicosatetraenoic acid | LMFA03040001 |
| 6R,15R-LXA5 | 15-epi-lipoxin A4 | (5S,6R,7E,9E,11Z,13E,15R)-5,6,15-trihydroxyicosa-7,9,11,13-tetraenoic acid | LMFA03040010 |
| 6S-LXA4 | epi-Lipoxin A4 | 5S,6S,15S-trihydroxy-7E,9E,11Z,13E-eicosatetraenoic acid | LMFA03040003 |
| LXA5 | LXA5 | 5S,6R,15S-trihydroxy-7E,9E,11Z,13E,17Z-eicosapentaenoic acid | No LM_ID in LIPID MAPS |
| LXB4 | Lipoxin B4 | 5S,14R,15S-trihydroxy-6E,8Z,10E,12E-eicosatetraenoic acid | LMFA03040002 |
| Resolvin E1 | Resolvin-E1 | 5S,12R,18R-trihydroxy-6Z,8E,10E,14Z,16E-eicosapentaenoic acid | LMFA03070019 |
| Resolvin D1 | Resolvin-D1 | 7S,8R,17S-trihydroxy-4Z,9E,11E,13Z,15E,19Z-docosahexaenoic acid | LMFA04000006 |
| 7,17 dHDPA | 7(S),17(S)-hydroxy DPA | 7,17-dihydroxy-8E,10Z,13Z,15E,19Z-docosapentaenoic acid | No LM_ID in LIPID MAPS |
| PDX | Protectin DX | 10S,17S-dihydroxy-4Z,7Z,11E,13Z,15E,19Z-docosahexaenoic acid | LMFA04040003 |
| 8,15-diHETE | 8,15-DiHETE | (5Z,9E,11Z,13E)-8,15-dihydroxyicosa-5,9,11,13-tetraenoic acid | LMFA03060109 |
| 15-HETE | 15S-HETE | 15S-hydroxy-5Z,8Z,11Z,13E-eicosatetraenoic acid | LMFA03060001 |
| 15-HEPE | (+/-) 15-HEPE | (+/-)-15-hydroxy-5Z,8Z,11Z,13E,17Z-eicosapentaenoic acid | LMFA03070032 |
| 17 HDoHE | (+/-)-17-HDoHE | (+/-)-17-hydroxy-4Z,7Z,10Z,13Z,15E,19Z-docosahexaenoic acid | LMFA04000032 |
| 13-HODE | 13(R)-HODE | 13R-hydroxy-9Z,11E-octadecadienoic acid | LMFA02000035 |
|  | 13(S)-HODE | 13S-hydroxy-9Z,11E-octadecadienoic acid | LMFA02000228 |
| 13-HOTrE | 13(S)-HOTrE | 13S-hydroxy-9Z,11E,15Z-octadecatrienoic acid | LMFA02000051 |
| 13-HOTrE(y) | 13(S)-HOTrE(γ) | 13S-​hydroxy-​6Z,​9Z,​11E-​octadecatrienoic acid | LMFA01050145 |
| 15-HETrE | 15(S)-HETrE | 15S-hydroxy-8Z,11Z,13E-eicosatrienoic acid | LMFA03050007 |
| 8-HETE | 8S-HETE | 8S-hydroxy-5Z,9E,11Z,14Z-eicosatetraenoic acid | LMFA03060006 |
| 8-HEPE | (+/-) 8-HEPE | (+/-)-8-hydroxy-5Z,9E,11Z,14Z,17Z-eicosapentaenoic acid | LMFA03070028 |
| 10 HDoHE | (+/-)-10-HDoHE | (+/-)-10-hydroxy-4Z,7Z,11E,13Z,16Z,19Z-docosahexaenoic acid | LMFA04000027 |
| 8-HETrE | 8(S)-HETrE | 8S-hydroxy-9E,11Z,14Z-eicosatrienoic acid | LMFA03050011 |
| 12-HETE | 12R-HETE | 12R-hydroxy-5Z,8Z,10E,14Z-eicosatetraenoic acid | LMFA03060008 |
|  | 12S-HETE | 12S-hydroxy-5Z,8Z,10E,14Z-eicosatetraenoic acid | LMFA03060007 |
| 12-HEPE | (+/-) 12-HEPE | (+/-)-12-hydroxy-5Z,8Z,10E,14Z,17Z-eicosapentaenoic acid | LMFA03070031 |
| 14 HDoHE | (+/-)-14-HDoHE | (+/-)-14-hydroxy-4Z,7Z,10Z,12E,16Z,19Z-docosahexaenoic acid | LMFA04000030 |
| 11 HDoHE | (+/-)-11-HDoHE | (+/-)-11-hydroxy-4Z,7Z,9E,13Z,16Z,19Z-docosahexaenoic acid | LMFA04000028 |
| 9-HODE | 9(R)-HODE | 9R-hydroxy-10E,12Z-octadecadienoic acid | LMFA02000036 |
|  | 9(S)-HODE | 9S-hydroxy-10E,12Z-octadecadienoic acid | LMFA02000188 |
| 5-oxoETE | 5-Oxo-ETE | 5-oxo-6E,8Z,11Z,14Z-eicosatetraenoic acid | LMFA03060011 |
| 12-oxoETE | 12-oxo-ETE | 12-oxo-5Z,8Z,10E,14Z-eicosatetraenoic acid | LMFA03060019 |
| 15-oxoETE | 15-Oxo-ETE | 15-oxo-5Z,8Z,11Z,13E-eicosatetraenoic acid | LMFA03060051 |
| 9-oxoODE | 9-OxoODE | 9-oxo-10E,12Z-octadecadienoic acid | LMFA02000274 |
| 13-oxoODE | 13-OxoODE | 13-keto-9Z,11E-octadecadienoic acid | LMFA02000016 |
| 15-oxoEDE | 15-Oxo-EDE | 15-oxo-11Z,13E-eicosadienoic acid | LMFA01060073 |
| 20-HETE | 20-HETE | 20-hydroxy-5Z,8Z,11Z,14Z-eicosatetraenoic acid | LMFA03060009 |
| 19-HETE | 19-HETE | (5Z,8Z,11Z,14Z)-19-hydroxy-5,8,11,14-icosatetraenoic acid | LMFA03060106 |
| 18-HETE | 18-HETE | 18-hydroxy-5Z,8Z,11Z,14Z-eicosatetraenoic acid | LMFA03060092 |
| 17-HETE | 17-HETE | 17-hydroxy-5Z,8Z,11Z,14Z-eicosatetraenoic acid | LMFA03060091 |
| 16-HETE | 16-HETE | (5Z,8Z,11Z,14Z)-16-hydroxyicosa-5,8,11,14-tetraenoic acid | LMFA03060105 |
| 18-HEPE | (+/-) 18-HEPE | (+/-)-18-hydroxy-5Z,8Z,11Z,14Z,16E-eicosapentaenoic acid | LMFA03070033 |
| 5,6-EET | (+/-)5,6-EpETrE | 5,6-epoxy-8Z,11Z,14Z-eicosatrienoic acid | LMFA03080002 |
| 8,9-EET | (+/-)8,9-EpETrE | 8,9-epoxy-5Z,11Z,14Z-eicosatrienoic acid | LMFA03080003 |
| 11,12-EET | (+/-)11,12-EpETrE | 11,12-epoxy-5Z,8Z,14Z-eicosatrienoic acid | LMFA03080004 |
| 14,15-EET | (+/-)14,15-EpETrE | 14,15-epoxy-5Z,8Z,11Z-eicosatrienoic acid | LMFA03080005 |
| 14(15)-EpETE | 14(15)-EpETE | (+/-)-14(15)-epoxy-5Z,8Z,11Z,17Z-eicosatetraenoic acid | LMFA03000003 |
| 17(18)-EpETE | 17(18)-EpETE | (+/-)-17(18)-epoxy-5Z,8Z,11Z,14Z-eicosatetraenoic acid | LMFA03000004 |
| 16(17)-EpDPE | 16(17) EpDPE | (+/-)-16(17)-epoxy-4Z,7Z,10Z,13Z,19Z-docosapentaenoic acid | LMFA04000037 |
| 19(20)-EpDPE | 19(20)-EpDPE | (+/-)-19(20)-epoxy-4Z,7Z,10Z,13Z,16Z-docosapentaenoic acid | LMFA04000038 |
| 19,20-DiHDPA | 19,20-DiHDPE | (+/-)-19,20-dihydroxy-4Z,7Z,10Z,13Z,16Z-docosapentaenoic acid | LMFA04000043 |
| 9,10-EpOME | 9(10)-EpOME | 9,10-epoxy-12Z-octadecenoic acid | LMFA02000037 |
| 12,13-EpOME | 12(13)-EpOME | (+/-)-12(13)-epoxy-9Z-octadecenoic acid | LMFA02000038 |
| 5,6-diHETrE | (+/-)5,6-DiHETrE | 5,6-dihydroxy-8Z,11Z,14Z-eicosatrienoic acid | LMFA03050004 |
| 8,9-diHETrE | (+/-)8,9-DiHETrE | 8,9-dihydroxy-5Z,11Z,14Z-eicosatrienoic acid | LMFA03050006 |
| 11,12-diHETrE | (+/-)11,12-DiHETrE | 11,12-dihydroxy-5Z,8Z,14Z-eicosatrienoic acid | LMFA03050008 |
| 14,15-diHETrE | (+/-)14,15-DiHETrE | 14,15-dihydroxy-5Z,8Z,11Z-eicosatrienoic acid | LMFA03050010 |
| 9,10-diHOME | 9,10-di-HOME | 9(10)-​dihydroxy-​12Z-​octadecenoic acid | LMFA01050350 |
| 12,13-diHOME | 12,13-DiHOME | 12,13-dihydroxy-9Z-octadecenoic acid | LMFA02000230 |
| Arachidonic Acid | Arachidonic acid | 5Z,8Z,11Z,14Z-eicosatetraenoic acid | LMFA01030001 |
| Adrenic Acid | Adrenic acid | 7,10,13,16-docosatetraenoic acid | LMFA04000050 |
| EPA | Eicosapentaenoic acid | 5Z,8Z,11Z,14Z,17Z-eicosapentaenoic acid | LMFA01030759 |
| DHA | Docosahexaenoic acid | 4Z,7Z,10Z,13Z,16Z,19Z-docosahexaenoic acid | LMFA01030185 |
| 20cooh AA | 20-carboxy-AA | 5Z,8Z,11Z,14Z-Eicosatetraenedioic acid | LMFA01170034 |
| 17k DPA | 17-oxo-DPA | 7Z,10Z,13Z,15E,19Z-17-oxo-docosapentaenoic acid | No LM_ID in LIPID MAPS |
| 2,3 dinor TXB2 | 2,3-Dinor-TXB2 | 9S,11,15S-trihydroxy-2,3-dinor-thromboxa-5Z,13E-dien-1-oic acid | LMFA03030003 |
| 11d-TXB2 | 11-dehydro-TXB2 | 9S,15S-dihydroxy-11-oxo-thromboxa-5Z,13E-dien-1-oic acid | LMFA03030004 |
| 2,3 dinor 8-iso PGF2a | 2,3-Dinor-8-iso-PGF2alpha | 9S,11R,15S-trihydroxy-2,3-dinor-5Z,13E-prostadienoic acid-cyclo[8S,12R] | LMFA03110010 |
| 2,3 dinor-6k PGF1a | 2,3-dinor, 6-keto-PGF1a | 6-oxo-9S,11R,15S-trihydroxy-2,3-dinor-13E-prostaenoic acid | LMFA03010089 |
| 8-iso PGF3a | 8-iso-PGF3alpha | 9S,11R,15S-trihydroxy-5Z,13E,17Z-prostatrienoic acid-cyclo[8S,12R] | LMFA03110007 |
| 8-iso-15k PGF2b | 8-iso-15-keto Prostaglandin F2β | 9β,11α-dihydroxy-15-oxo-(8β)-prosta-5Z,13E-dien-1-oic acid | No LM_ID in LIPID MAPS |
| 9-Nitrooleate | 9-Nitrooleate | 9-nitro-9E-octadecenoic acid | LMFA01120004 |
| 10-Nitrooleate | 10-Nitrooleate | 10-nitro-9E-octadecenoic acid | LMFA01120003 |
| Tetanor-PGDM | Tetranor-PGDM | 9S-hydroxy-11,15-dioxo-2,3,4,5-tetranor-prostan-1,20-dioic acid | LMFA03010221 |
| Maresin 1 | Maresin 1 | 7R,14S-dihydroxy-4Z,8E,10E,12Z,16Z,19Z-docosahexaenoic acid | LMFA04050001 |
| Resolvin D2 | Resolvin D2 | 7S,16R,17S-trihydroxy-4Z,8E,10Z,12E,14E,19Z-docosahexaenoic acid | LMFA04030001 |
| Resolvin D3 | Resolvin D3 | 4S,10,17S-trihydroxy-5E,7E,9E,13Z,15E,19Z-docosahexaenoic acid | LMFA04030012 |
| Resolvin D5 | Resolvin D5 | 7S,17S-dihydroxy-4Z,8E,10Z,13Z,15E,19Z-docosahexaenoic acid | LMFA04030003 |
| Resolvin E2 | Resolvin E2 | 5S,18R-dihydroxy-6E,8Z,11Z,14Z,16E-eicosapentaenoic acid | LMFA03140011 |
| Resolvin E4 | Resolvin E4 | 5S,15S-dihydroxy-6E,8Z,11Z,13E,17Z-eicosapentaenoic acid | LMFA03140013 |
| Resolvin D2 (DPA N3) | Resolvin D2 (DPA N3) | 7S,16R,17S-trihydroxy-8E,10Z,12E,14E,19Z- docosapentaenoic acid | No LM_ID in LIPID MAPS |
| Maresin 2 | Maresin 2 | 13R,14S-dihydroxy-4Z,7Z,9E,11E,16Z,19Z-docosahexaenoic acid | LMFA04050004 |
| Neuroprotectin D1 | Neuroprotectin D1 | 10R,17S-dihydroxy-4Z,7Z,11E,13E,15Z,19Z-docosahexaenoic acid | LMFA04040001 |
| 17(R)-Protectin D1 | 17(R)-Protectin D1 | 10R,17R-dihydroxy-4Z,7Z,11E,13E,15Z,19Z-docosahexaenoic acid | No LM_ID in LIPID MAPS |

LOD = limit of detection.
