# Supplementary material for: Urinary prostaglandin metabolites as biomarkers for human labour: Insights into future predictors
Source: PLoS One. 2025 Jul 14;20(7):e0315484. doi: 10.1371/journal.pone.0315484 (PMC12258607; doi:10.1371/journal.pone.0315484)
Supplement: S4 Appendix — (DOCX) [file pone.0315484.s004.docx]

Analyses excluding n=4 participants with PPROM in the TPTL-PTD group.

| **Eicosanoid** | **Analysis** | **TPTL-PTD vs TPTL-TD (mean)** | **p-value** | **Association between PGs and cervical index** | **p-value** | **Association between PGs and time to delivery** | **p-value** |
| --- | --- | --- | --- | --- | --- | --- | --- |
| **PGFM** | All samples (n=43 TPTL-PTD) | 10.46 vs 10.35 | p = 0.69 | R^2^ = 0.014, β = -0.09 | p = 0.33 | R^2^ = 0.046, β = -0.008 | p = 0.048 |
|  | Exclude n=4 PPROM (n=39 TPTL-PTD) | 10.51 vs 10.35 | p = 0.57 | R^2^ = 0.018, β = -0.07 | p = 0.28 | R^2^ = 0.055, β = -0.009 | p = 0.034 |
| **PGEM** | All samples (n=43 TPTL-PTD) | 10.23 vs 10.00 | p = 0.46 | R^2^ <0.001, β = -0.012 | p = 0.86 | R^2^ = 0.013, β = -0.005 | p = 0.30 |
|  | Exclude n=4 PPROM (n=39 TPTL-PTD) | 10.38 vs 10.00 | p = 0.22 | R^2^ = 0.001, β = -0.019 | p = 0.78 | R^2^ = 0.025, β = -0.007 | p = 0.15 |
| **t-PGDM** | All samples (n=43 TPTL-PTD) | 12.47 vs 12.70 | p = 0.29 | R^2^ = 0.081, β = 0.11 | p = 0.017 | R^2^ = 0.009, β = -0.003 | p = 0.37 |
|  | Exclude n=4 PPROM (n=39 TPTL-PTD) | 12.55 vs 12.70 | p = 0.50 | R^2^ = 0.077, β = 0.11 | p = 0.022 | R^2^ = 0.021, β = -0.005 | p = 0.19 |
| **8-isoprostane** | All samples (n=43 TPTL-PTD) | 10.81 vs 10.10 | p = 0.20 | R^2^ <0.001, β = -0.005 | p = 0.95 | R^2^ = 0.074, β = -0.015 | p = 0.017 |
|  | Exclude n=4 PPROM (n=39 TPTL-PTD) | 10.83 vs 10.10 | p = 0.25 | R^2^ <0.001, β = 0.002 | p = 0.97 | R^2^ = 0.076, β = -0.015 | p = 0.018 |
| **PGIM** | All samples (n=43 TPTL-PTD) | 14.44 vs 14.99 | p = 0.036 | R^2^ = 0.070, β = 0.12 | p = 0.035 | R^2^ = 0.062, β = 0.010 | p = 0.024 |
|  | Exclude n=4 PPROM (n=39 TPTL-PTD) | 14.50 vs 14.99 | p = 0.071 | R^2^ = 0.067, β = 0.12 | p = 0.039 | R^2^ = 0.053, β = 0.009 | p = 0.041 |
| **PGF2a** | All samples (n=43 TPTL-PTD) | 10.64 vs 10.60 | p = 0.86 | R^2^ <0.001, β = 0.010 | p = 0.86 | R^2^ = 0.065, β = -0.010 | p = 0.022 |
|  | Exclude n=4 PPROM (n=39 TPTL-PTD) | 10.75 vs 10.60 | p = 0.56 | R^2^ <0.001, β = 0.008 | p = 0.89 | R^2^ = 0.092, β = -0.011 | p = 0.008 |

Red text indicates differences in significance between analyses.
