# Supplementary material for: Urinary prostaglandin metabolites as biomarkers for human labour: Insights into future predictors
Source: PLoS One. 2025 Jul 14;20(7):e0315484. doi: 10.1371/journal.pone.0315484 (PMC12258607; doi:10.1371/journal.pone.0315484)
Supplement: S5 Appendix — (PDF) [file pone.0315484.s005.pdf]

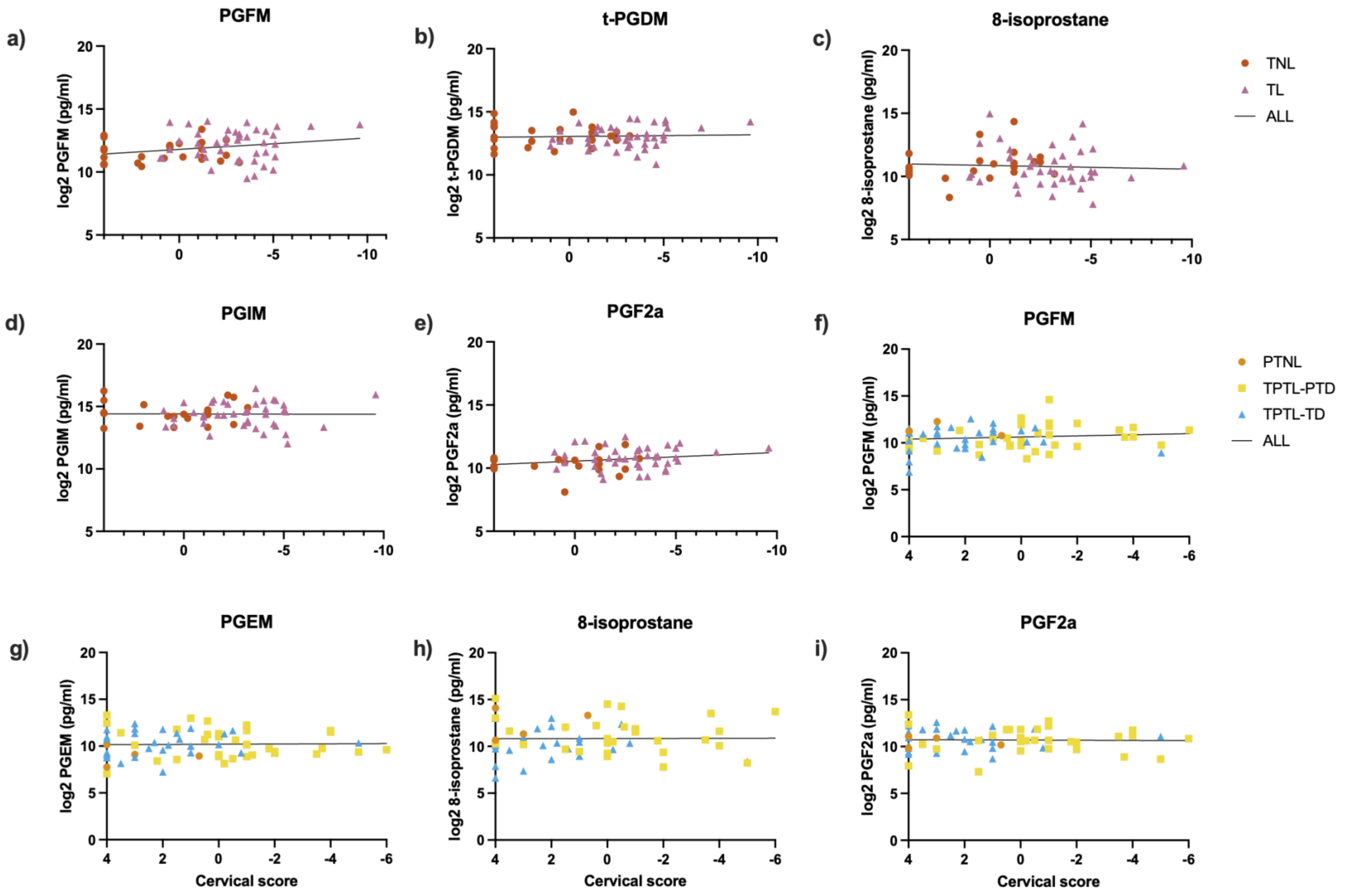
a)
PGFM
log2 PGFM (pg/ml)
b)
t-PGDM
log2 t-PGDM (pg/ml)
c)
8-isoprostane
log2 8-isoprostane (pg/ml)
TNL
TL
ALL
d)
PGIM
log2 PGIM (pg/ml)
e)
PGF2a
log2 PGF2a (pg/ml)
f)
PGFM
log2 PGFM (pg/ml)
PTNL
TPTL-PTD
TPTL-TD
ALL
g)
PGEM
log2 PGEM (pg/ml)
h)
8-isoprostane
log2 8-isoprostane (pg/ml)
i)
PGF2a
log2 PGF2a (pg/ml)
Cervical score

Supplemental Fig 2. Urinary prostaglandin metabolite levels not associated with cervical score in term or preterm pregnancy. Analyzed by linear regression. a) PGFM: R^2 = 0.056, β = -0.090, p = 0.055; b) t-PGDM: R^2 = 0.003, β = -0.014, p = 0.69; c) 8-isoprostane: R^2 = 0.003, β = 0.030, p = 0.65; d) PGIM: R^2 <0.001, β = 0.003, p = 0.95; e) $PGF_{2\alpha}$: R^2 = 0.053, β = -0.068, p = 0.072; Regression equations represent all samples from the TL and TNL groups together. f) PGFM: R^2 = 0.014, β = -0.09, p = 0.33; g) PGEM: R^2 <0.001, β = -0.012, p = 0.86; h) 8-isoprostane: R^2 <0.001, β = -0.005, p = 0.95; i) $PGF_{2\alpha}$: R^2 <0.001, β = 0.010, p = 0.86. Regression equations represent all samples from PTNL, TPTL-PTD and TPTL-TD groups together. TL = term labour, TNL = term non-labour. PTNL = preterm non-labour controls, TPTL-PTD = threatened preterm labour-preterm delivery, TPTL-TD = threatened preterm labour–term delivery.
